# *Mmu-miR-185* depletion promotes osteogenic differentiation and suppresses bone loss in osteoporosis through the Bgn-mediated BMP/Smad pathway

**DOI:** 10.1038/s41419-019-1428-1

**Published:** 2019-02-20

**Authors:** Qi Cui, Jinhao Xing, Miao Yu, Yue Wang, Jian Xu, Yajuan Gu, Xu Nan, Wenping Ma, Hao Liu, Hongshan Zhao

**Affiliations:** 1 Department of Medical Genetics, Peking University School of Basic Medical Sciences, 100191 Beijing, China; 2 Peking University Center for Human Disease Genomics, 100191 Beijing, China; 3 Department of Prosthodontics, Peking University School and Hospital of Stomatology and National Clinical Research Center for Oral Diseases National Engineering Laboratory for Digital and Material Technology of Stomatology and Beijing Key Laboratory of Digital Stomatology, 100081 Beijing, China; 4 Department of Anatomy, Histology and Embryology, Peking University School of Basic Medical Sciences, 100191 Beijing, China; 5 The Central Laboratory, Peking University School and Hospital of Stomatology and National Clinical Research Center for Oral Diseases and National Engineering Laboratory for Digital and Material Technology of Stomatology and Beijing Key Laboratory of Digital Stomatology, 100081 Beijing, China

## Abstract

MicroRNAs (miRs) play an essential role in the regulation of bone formation and homeostasis. *miR-185* has been reported to negatively regulate osteogenesis in vitro. However, whether it has an impact on in vivo bone homeostasis remains unknown. Here, we demonstrated that primary osteoblasts and mesenchymal stem cells derived from *miR-185*-knockout (KO) mice exhibited enhanced osteogenesis. Further, we constructed an ovariectomized mouse model to investigate the role of *miR-185* during osteoporosis. Micro-computed tomography revealed an increased bone volume in KO compared to wild-type mice 6 weeks after surgery, indicating redundant bone formation after *miR-185* depletion. Dual-luciferase reporter assays identified biglycan (Bgn), which promotes bone formation through the BMP/Smad pathway, as the direct target of *miR-185*. Taken together, these findings indicate that blocking *miR-185* expression increases bone formation during osteoporosis, which may partly occur through the regulation of Bgn expression and BMP/Smad signaling.

## Introduction

Bone homeostasis is a dynamic balance that includes bone formation by osteoblasts and bone resorption by osteoclasts. Osteoporosis, characterized by bone mass loss and micro-architectural deterioration, is a common skeletal disease resulting in high susceptibility to fracture. Studies published since the 1960s have shown a relationship between osteoporosis and menopause^1^. Estrogen deficiency during menopause decreases bone formation, while osteoclastic resorption activity is accelerated, leading to bone loss. Bilateral ovariectomy (OVX), a classic method for constructing animal models of osteoporosis, is widely used in studies of bone metabolism.

MicroRNAs (miRs) are evolutionarily conserved endogenous non-coding RNAs approximately 21–23 nucleotides in length. They recruit the RNA-induced silencing complex to the complementary sequences of their target messenger RNAs (mRNAs), causing mRNA degradation or repressing translation to interfere with targeted gene expression^2,3^. They play a crucial role in regulating bone formation and remodeling^4,5^.

In a previous study, we found that *mmu*-*miR-185*-5p was upregulated by mutant RUNT-related transcription factor 2 (Runx2), and in vitro studies demonstrated that *miR-185* inhibited osteogenesis in MC3T3-E1 cells^6^. Nonetheless, the functions of *miR-185* in bone homeostasis in vivo remain underexplored.

Here, we constructed *miR-185*-knockout (KO) mice using CRISPR/Cas9 genome editing technology and examined bone formation in KO mice. Primary osteoblasts and mesenchymal stem cells (MSCs) derived from KO mice showed stronger osteogenic ability than those of wild-type (WT) mice. Moreover, *miR-185* depletion attenuated the osteoporotic symptoms caused by lack of estrogen in an OVX mouse model. We also found that *miR-185* regulated the expression of biglycan (Bgn), in part through which the BMP/Smad signaling pathway was also affected. These findings provide new insights into the regulatory role of miRNAs in bone formation.

## Materials and methods

### Antibodies and reagents

Antibodies to GAPDH (Sungene Biotech, KM9002), Alp (Abcam, ab108337), osterix (Osx) (Bioss, bs-1110R), Dlx2 (Proteintech, 26244-1-AP), Bgn (Proteintech, 16409-1-AP), Bmp2 (Proteintech, 18933-1-AP), Smad1 (Proteintech, 10429-1-AP), and phospho-Smad1/5/8 (CST,13820) were purchased commercially.

### Animals

#### Generation of *mmu-miR-185* knockout mice

*Mmu-miR-185*-KO)mice were generated by the Shanghai Model Organisms Center (China) using CRISPR/Cas9 technology. Genotyping was performed using DNAs extracted from mice tails, and KO mice were identified with a loss of 104 bp in genomes, which covers the whole precursor-miR-185 encoding sequence. All mice used in the study were housed and bred in the Department of Laboratory Animal Science, Peking University Health Sciences Center (Beijing, China). Mice used had free access to clean water and food, and were maintained under a 12 h light/dark cycle. All animal experiments were performed in accordance with the NIH guidelines and were approved by Biomedical Research Ethics Committee of Peking University. PCR genotyping was carried out using specific primers as follows (5′–3′):

F: AGGGAATGGCTAGGGTTTGC.

R: TACTGGGTAGGACCTCTGGC.

#### OVX mouse model

WT or KO female mice aged 8 weeks were randomly divided into four groups: WT mice subject to sham (WT Sham) or ovariectomy (WT OVX) operation, and KO mice subject to sham (KO Sham) or ovariectomy (KO OVX) operation. For the OVX groups, mice were anesthetized, and bilateral ovariectomies were conducted through small dorsal incisions. The sham operations were given on sham groups as negative controls (NCs). Six weeks after operation, all the mice were humanely euthanized, and bone samples were harvested and fixed.

For miR-185 restoration experiments, the mice were injected intravenously with miR-185 agomir or NC as described previously^7,8^. Briefly, mice underwent three injections in the first week after OVX (80 mg/kg/day), and another injection on the first day of the fourth week. Bone samples were harvested 6 weeks after OVX.

### Cell culture and transfection

Primary osteoblasts were derived as described previously^9^. Briefly, 1- to 3-day-old neonatal mice were sacrificed, and the hemicalvaria was harvested under sterile environment. After cleaning of adherent soft tissue, the calvaria was digested with collagenase and cells were plated into the α-minimum essential medium (αMEM) containing 10% fetal bovine serum (FBS) and antibiotics. For osteoblastic differentiation experiments, the medium was changed into fresh osteoblast induction medium (OIM), that is, αMEM supplemented with 10 mM β-glycerophosphate (Sigma-Aldrich, G9422), 50 μg/ml ascorbic acid (Sigma-Aldrich, A8960), and 100 nm dexamethasone (Sigma-Aldrich, D4902). The medium was changed every 3 days throughout the experiments and cells were harvested at indicated time points.

For the culture of MSCs, bone marrows were flushed from femurs and tibias of mice under aseptic conditions and resuspended in αMEM. The medium was changed every 3 days and the floating cells were removed. The medium was replaced by OIM for osteoblast induction experiments.

The human embryonic kidney 293T (HEK293T) cells were cultured in Dulbecco’s modified Eagle’s medium (Invitrogen, Carlsbad, CA, USA) and the MC3T3-E1 cells were cultured in αMEM basic medium, both supplemented with 100 U/ml penicillin, 0.1 mg/ml streptomycin, and 10% FBS (ExCell, FSP500). The miRNA mimic, inhibitor, and NC RNA were purchased commercially (Gene Pharma Inc., Shanghai, China). MiRNA mimic and small interfering RNA (siRNA) transfection was performed using Lipofectamine 3000 reagent (Life Technologies, Carlsbad, CA, USA) according to the manufacturer’s instructions.

### Real-time PCR

TRIzol reagent (Invitrogen Life Technologies, 15596026) was used to extract total RNA from cells and tissues. After concentration determination, equal amounts of RNAs (0.5–1 μg) were reverse transcripted into complementary DNA (cDNA) using the RT reagent kit (TAKARA, RR037A) according to the manufacturer’s instruction. For extraction of RNA in the bone, the bone marrow was flushed out, and the bone tissue were grinded with liquid nitrogen before the addition of TRIzol.

Real-time PCR reactions were performed using the AceQ qPCR SYBR Green Master Mix (Vazyme Biotech, Q131-02) in a 20-μl reaction mixture. Each reaction was performed in triplicate. The expression levels of mRNA were quantified and normalized to β-actin using the 2−ΔΔCt method. The primer sequences are shown as follows (5′–3′):

Osx (F: ATGGCGTCCTCTCTGCTTG; R: TGAAAGGTCAGCGTATGGCTT).

Osteocalcin (OC) (F: CTGACCTCACAGATCCCAAGC; R: TGGTCTGATAGCTCGTCACAAG).

Collagen type 1α 1 (Col1a1) (F: CTGGCGGTTCAGGTCCAAT; R: TTCCAGGCAATCCACGAGC).

Dlx2 (F: GTGGCTGATATGCACTCGACC; R: GCTGGTTGGTGTAGTAGCTGC).

β-Actin (F: GGCTGTATTCCCCTCCATCG; R:CCAGTTGGTAACAATGCCATGT).

### Western blot

Cellular proteins were extracted using RIPA Lysis Buffer (Beyotime Biotechnology, Shanghai, China) supplemented with proteinase inhibitor cocktail (Roche Diagnostics, Berlin, Germany). BCA protein assay kit (Beyotime Biotechnology, PC0020, Shanghai, China) was used for determining the protein concentrations. For extraction of protein in bone, the bone marrow was flushed out, and the bone tissue were grinded with liquid nitrogen before the addition of RIPA. Equal amounts of proteins were subjected to 10–15% sodium dodecyl sulfate-polyacrylamide gel electrophoresis and transferred onto nitrocellulose membranes. Membranes were then blocked with 5% bovine serum albumin or nonfat milk for 1 h, and incubated with the primary antibody overnight at 4 °C. After washing twice with TBST, the membranes were incubated with secondary antibodies (ZSGB BIO, ZB2305, ZB2301) for 1 h at room temperature. Protein bands were detected by ECL detection kit (Thermo Scientific, 34077) and imaged.

### Cell proliferation assay

Cell Counting Kit-8 (CCK-8) detection kit (Dojindo, Japan) was used to analyze the cell proliferation ability. Briefly speaking, 4 × 10^3^ cells were seeded into each well of a 96-well plate and cultured in αMEM containing 10% FBS and antibiotics. Ten microliters of CCK-8 was added, and absorbance values were measured 2 h later at 450 nm.

### Alkaline phosphatase and Alizarin Red S staining and activity determination

A total of 2 × 10^4^ cells were seeded per 24-well plate, and cultured in OIM for 7 or 14 days. Cells were washed twice with 1× phosphate-buffered saline, and fixed by ice-cold ethanol for 10 min. Alkaline phosphatase (ALP) staining buffer were prepared according to the manufacturer's instructions (CWBIO, cw0051), and added to the cells. After incubation for 10–15 min at room temperature, cells were washed and photographed under the microscope.

For ALP activity determination, the ALP assay kit (Njjcbio, A059-2, Nanjing, China) was used. Cells were lysed by 1% Triton for 30 min at 4 °C, and the lysates were added into the reaction buffer. After incubation at 37 °C for 15 min, the coloration liquid was added and the absorbance at 520 nm of each sample was measured.

Alizarin Red S staining was carried out in 24-well plates. The osteoblasts were treated with OIM for 14 and 21 days, and fixed with 70% ethanol. Cells were incubated with 0.5% Alizarin Red solution for 1 h at room temperature and images were taken under the microscope.

For matrix mineralization quantification, stained cells were incubated with 100 mM cetylpyridinium chloride for 1 h. The calcium-bound Alizarin Red S was eluted and measured at OD570nm by the spectrophotometer.

### Bone mineral density and morphometry

Mouse femurs were dissected free of soft tissue, fixed in 4% paraformaldehyde for 24 h, and scanned under micro-computed tomography (μCT). Scanning parameters were set as follows: voltage of 60 kV, current of 220 μA, exposure time of 1500 ms, and effective pixel size of 8.89 μm. Bone mineral density (BMD, mg/cm^3^) was calculated, and the morphometry of cortical and trabecular bones were evaluated using high-resolution Inveon microtomography (Siemens, Munich, Germany).

For further quantitative analysis, volumes of interest (VOI) of the trabecular bones were established, which included the 1–2 mm region distal to the proximal epiphysis. Parameters such as bone volume/total volume (BV/TV), bone surface area/BV (BSA/BV), trabecular thickness (Tb. Th), trabecular bone number (Tb. N), trabecular spacing (Tb. Sp), and trabecular pattern factor (Tb. Pattern Factor) were analyzed by Inveon Research Workplace.

Histological analysis was performed as previously described^10^. Briefly, the femurs were fixed with 4% paraformaldehyde followed by decalcification with 10% EDTA (pH 7.4). The bone samples were dehydrated followed by paraffin embedding. Slices (4 μm thick) were prepared and hematoxylin and eosin (H&E), Masson, Fast green staining were carried out.

### Dual-luciferase reporter assay

psiCHECK™-2 Vector could detect both the firefly and Renilla luciferase activities. psiCHECK2-Bgn plasmids were constructed containing either WT or mutant 3′-untranslated region (UTR) sequence of Bgn mRNA. The plasmids were transfected into HEK293T cells with miR-185 mimic or NC using Lipofectamine 3000 reagents. Cells were harvested 48 h after transfection. The activities of firefly and Renilla luciferases were measured sequentially by a luminometer (Glomax; Promega) using the Dual-Luciferase Reporter Assay (Promega). Relative luciferase activities were quantified by normalizing Renilla luciferase values to firefly values.

### Statistical analysis

All statistical data in this study was representative of three or more independent experiments and presented as mean ± SD. Differences between groups were detected using the Student’s *t* test. Two-sided *p* values <0.05 were considered statistically significant.

## Result

### Generation of *mmu-miR-185* –/– mice

**Fig. 1:**
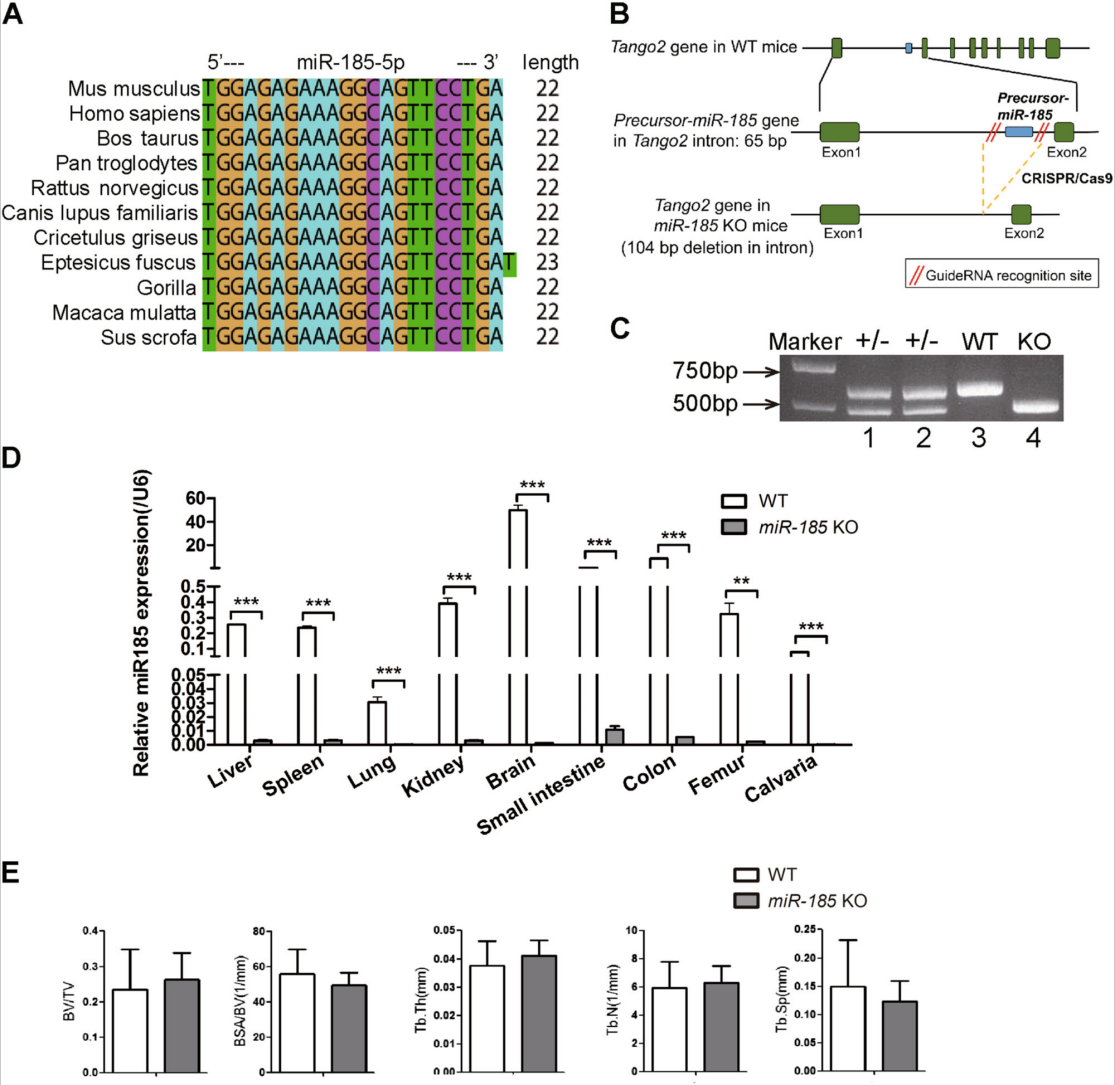
Generation of *mmu-miR-185*−/− mice. **a** Analysis of miR-185 conservation in different species. **b** Scheme used to generate *miR-185* knockout mice. **c** Genotyping of transgenic mice by PCR with DNA extracted from mouse tail. Expected band sizes: *miR-185* knockout (KO) 512 bp, wild type (WT) 616 bp, heterozygous showed two bands. **d** Representative real-time PCR result showed endogenous miR-185 expression levels in different tissues of WT and KO mice (*n* = 3). **e** Micro-computed tomography (μCT) analysis of 6-week-old WT and KO male mice femur samples (*n* = 5). Bone volume/total volume (BV/TV), trabecular bone surface/bone volume (BSA/BV), trabecular bone thickness (Tb. Th), trabecular bone number (Tb. N), and trabecular bone space (Tb. Sp). Data were shown as mean ± S.D (**P < 0.01 ***P < 0.001)

Conservation analyses showed that *miR-185* is highly conserved among species (Fig. 1a). *miR-185*-KO) mice were generated using CRISPR/Cas9 technology, which caused the deletion of the full-length coding sequence of *pre-miR-185* (Fig. 1b, S1A). The genotyping results for the mice used are shown in Fig. 1c and S1B. The deletion of *miR-185* in KO mice was also confirmed using real-time PCR, and hardly any *miR-185* expression was detected in tissues and organs of KO mice compared to those of WT mice (Fig. 1d). The depleted sequence was within intron 1 of the *Tango2* gene, and the real-time PCR results indicated that Tango2 expression in KO mice was unaffected (Fig. S1C).

*miR-185*-KO mice had a normal body weight and body length comparable with those of WT littermate control mice (data not shown), and did not exhibit spontaneous phenotypes. Bone samples were harvested and μCT showed normal trabecular bone morphometry in femurs of KO mice (Fig. 1e).

### *miR-185* silencing promotes primary osteoblast differentiation and mineralization

**Fig. 2:**
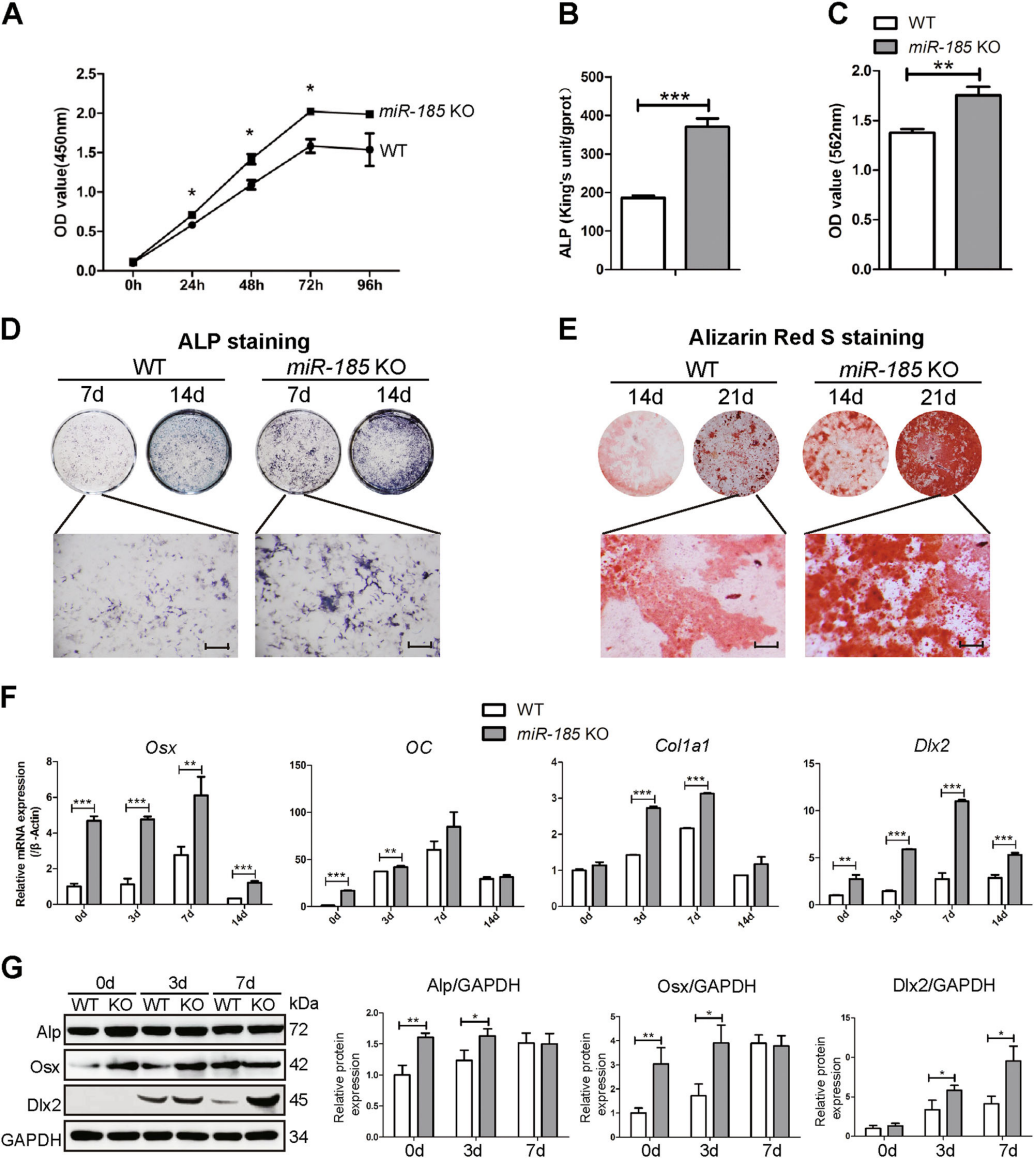
*MiR-185* silencing promotes primary osteoblast differentiation and mineralization. **a** Cell Counting Kit-8 (CCK-8) assay reflected cell proliferation of osteoblasts derived from wild-type (WT) or *miR-185*-knockout (KO) mice calvaria. **b** Alkaline phosphatase (ALP) activity determination in WT or *miR-185-*KO osteoblasts after cultured with osteoblast induction medium (OIM) for 7 days (*n* = 3). **c** Matrix mineralization was quantified in primary osteoblasts after induction in OIM for 14 days (*n* = 3). **d** Representative images of ALP staining of WT or *miR-185* KO cells after osteoblast induction for 7 or 14 days. Scale bar = 500 μm. **e** Representative images of Alizarin Red S staining in cells after osteoblast induction for 14 or 21 days. Scale bars = 500 μm. **f** The primary osteoblasts were cultured in OIM for indicated times. RNA in cells was extracted with TRIzol reagent, and the expression levels of osteoblast marker genes were quantified by real-time PCR (*n* = 3). **g** The protein levels of osteoblast marker genes in primary osteoblasts cultured with OIM for 0, 3, and 7 days were analyzed by western blot, and expressed as densitometry normalized to glyceraldehyde 3-phosphate dehydrogenase (GAPDH). Data were representative of at least three independent experiments, and were shown as the mean ± S.D (*P < 0.05, **P < 0.01, ***P < 0.001)

In a previous study, we demonstrated that *miR-185*-5p had an inhibitory role in MC3T3-E1 cell osteogenesis. To determine the effects of *miR-185* on primary osteoblast differentiation, we examined the osteogenic ability of osteoblasts derived from the calvaria of neonatal mice. As shown in Fig. 2a, primary osteoblasts of KO mice exhibited increased proliferative ability in CCK-8 assays. Meanwhile, after osteoblastic induction for 7 and 14 days, alkaline phosphatase (ALP) quantification assays indicated increased ALP activity in KO osteoblasts (Fig. 2b). ALP staining results also suggested significantly elevated ALP expression in KO cells (Fig. 2d).

To investigate whether *miR-185* is involved in terminal osteogenic differentiation, osteoblast mineralization was analyzed using Alizarin Red S staining. After induction for 14 and 21 days, the KO cells showed significantly increased mineralization (Fig. 2e). Alizarin Red S quantification also indicated enhanced mineralized matrix formation in *miR-185*-depleted cells (Fig. 2c).

Further, we examined the expression of osteoblast marker genes, and increased mRNA abundance of Osx, OC, and Col1a1 was detected in KO cells. Dlx2, identified as the target gene of *miR-185*^6^, was highly expressed in KO cells on days 0, 3, 7, and 14 of induction compared to expression levels in WT cells (Fig. 2f). Increased protein levels of ALP, Osx, and Dlx2 in KO cells were also detected by Western blotting at the indicated time points during osteoblastic induction (Fig. 2g). Taken together, these data suggest that primary osteoblasts with *miR-185* depletion showed increased differentiation and mineralization ability.

### *miR-185* silencing promotes the osteogenic function of MSCs

**Fig. 3:**
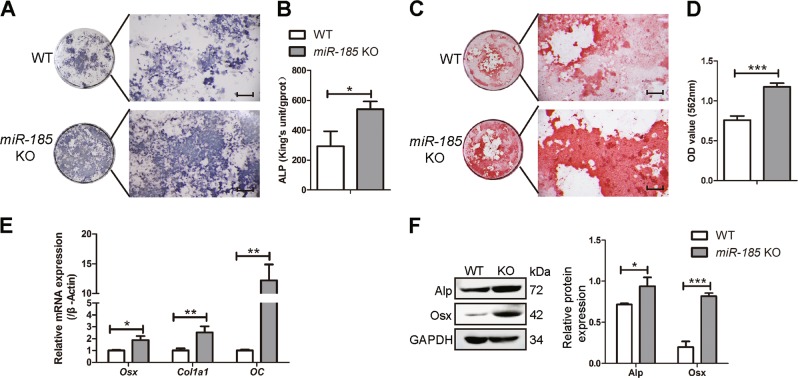
MiR-185 silencing promotes the osteogenic function of mesenchymal stem cells (MSCs). MSCs were derived from the bone marrow of wild-type (WT) or knockout (KO) mice, and induced with osteoblast induction medium (OIM) for osteogenic differentiation. **a** Alkaline phosphatase (ALP) staining in WT or KO MSCs after osteoblast induction for 7 days. Scale bar = 500 μm. **b** ALP activity determination in MSCs after osteogenic induction for 7 days. **c** Alizarin Red S Staining in MSCs after osteoblast induction for 21 days. Scale bar = 500 μm. **d** Matrix mineralization was quantified in MSCs after induction for 21 days. **e** Real-time PCR showed the messenger RNA (mRNA) expression levels of osterix (Osx), collagen type 1α 1 (Col1a1), and osteocalcin (OC) in MSCs after induction with OIM for 7 days. **f** The expression of osteoblast marker genes in MSCs cultured for 3 days in OIM was indicated by western blot. Data were shown as mean ± S.D (*P < 0.05, **P < 0.01, ***P < 0.001)

To further analyze the role of *miR-185* in regulating osteogenic differentiation of MSCs, ALP staining and quantification were performed. The results revealed increased expression and enhanced activity of ALP in KO MSCs 7 days after osteoblastic induction (Fig. 3a, b). Alizarin Red S staining was also increased in KO MSCs after induction for 21 days (Fig. 3c, d). The expression levels of osteogenic markers were analyzed, and upregulation of Osx, Col1a1, OC, and ALP was verified by real-time PCR and Western blotting (Fig. 3e, f). Taken together, MSCs showed enhanced osteogenic function in the absence of *miR-185*.

### *miR-185* depletion attenuates bone loss induced by estrogen deficiency

**Fig. 4:**
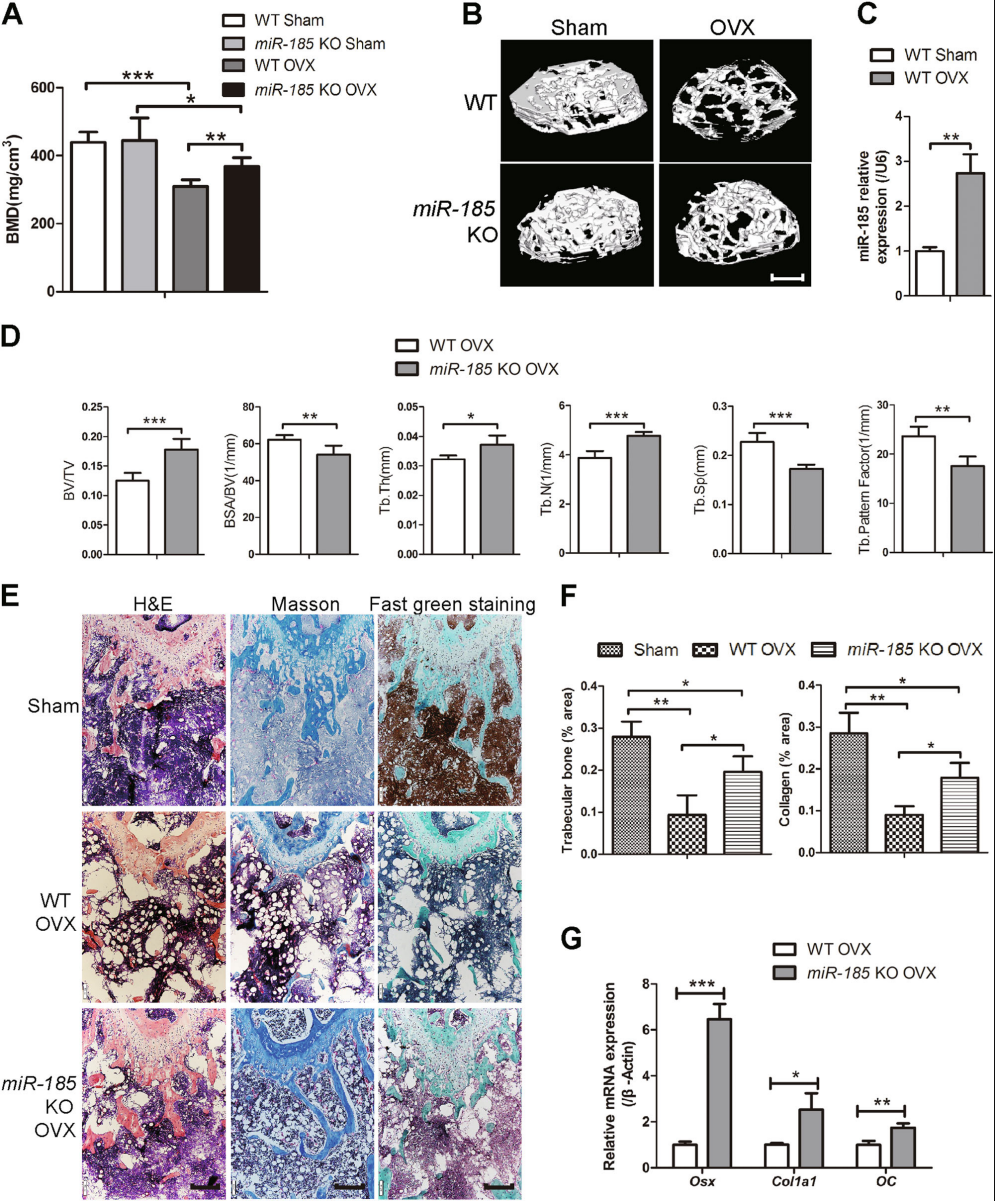
Depletion of *miR-185* attenuates bone loss in ovariectomized (OVX) mouse model. Femur samples were harvested 6 weeks after OVX or Sham operation. **a** The bone mineral density (BMD) values of mice femurs were calculated by micro-computed tomography (μCT) (*n* = 5). **b** Three-dimensional (3D) reconstructive images of trabecular bones in femur epiphysis. Scale bar = 400 μm. **c** Real-time PCR indicated the *miR-185* expressions in femurs of wild-type (WT) Sham and WT OVX groups. **d** The bone volume/total volume (BV/TV), trabecular bone surface/bone volume (BSA/BV), trabecular bone thickness (Tb. Th), trabecular bone number (Tb. N), trabecular bone space (Tb. Sp), and trabecular pattern factor (Tb. Pf) of femur epiphysis were calculated by μCT (*n* = 5). **e** Bone samples were decalcied and bone slices were prepared. Representative images of hematoxylin and eosin (H&E), Masson’s trichrome staining and Fast green staining were shown. Scale bar = 200 μm. **f** The relative area of trabecular bone and collagen were analyzed using Image J program (*n* = 3). **g** The femur bone tissue (removed of bone marrow) were grinded with liquid nitrogen, and the RNA was extracted. mRNA expression of osteoblast markers in OVX groups was determined by real-time PCR. Data were shown as mean ± S.D (*P < 0.05, **P < 0.01, ***P < 0.001)

To investigate the role of *miR-185* in the progression of osteoporosis, WT and KO mice were subject to either OVX or sham operation. Bones were harvested 6 weeks later, and μCT was conducted to analyze bone mass loss and bone microstructure destruction in femur epiphyses. BMD values were dramatically lower in the OVX groups than in the sham group, and 3D reconstruction images indicated that OVX mice had a reduced trabecular BV, confirming that effective estrogen depletion models were established (Fig. 4a, b). In WT mice, *miR-185* expression in femurs was upregulated after OVX surgery, as indicated by real-time PCR (Fig. 4c). Among OVX groups, the *miR-185* KO mice had higher BMD values than the WT mice. The BV/TV and Tb. N of femur epiphyses were significantly less reduced in KO mice than in WT mice, and μCT images also indicated greater Tb. Th. in KO than in WT mice. By contrast, BSA/BV, Tb. Sp., and Tb. Pattern Factor were lower in KO mice than in WT mice (Fig. 4d).

The suppression of osteoporosis was also confirmed by histological analyses. There was greater collagen staining in KO OVX mice femurs, as indicated by Masson’s trichrome staining. Fast green staining also revealed increased trabecular bone area in KO OVX mice (Fig. 4e, f). Further, real-time PCR indicated higher expression levels of Osx, Col1a1, and OC in KO bones than in WT bones (Fig. 4g). Taken together, these results indicate that *miR-185* depletion attenuates the osteoporosis phenotype in the OVX mouse model.

### *miR-185* restoration reverses the increased bone phenotype in KO mice after OVX

**Fig. 5:**
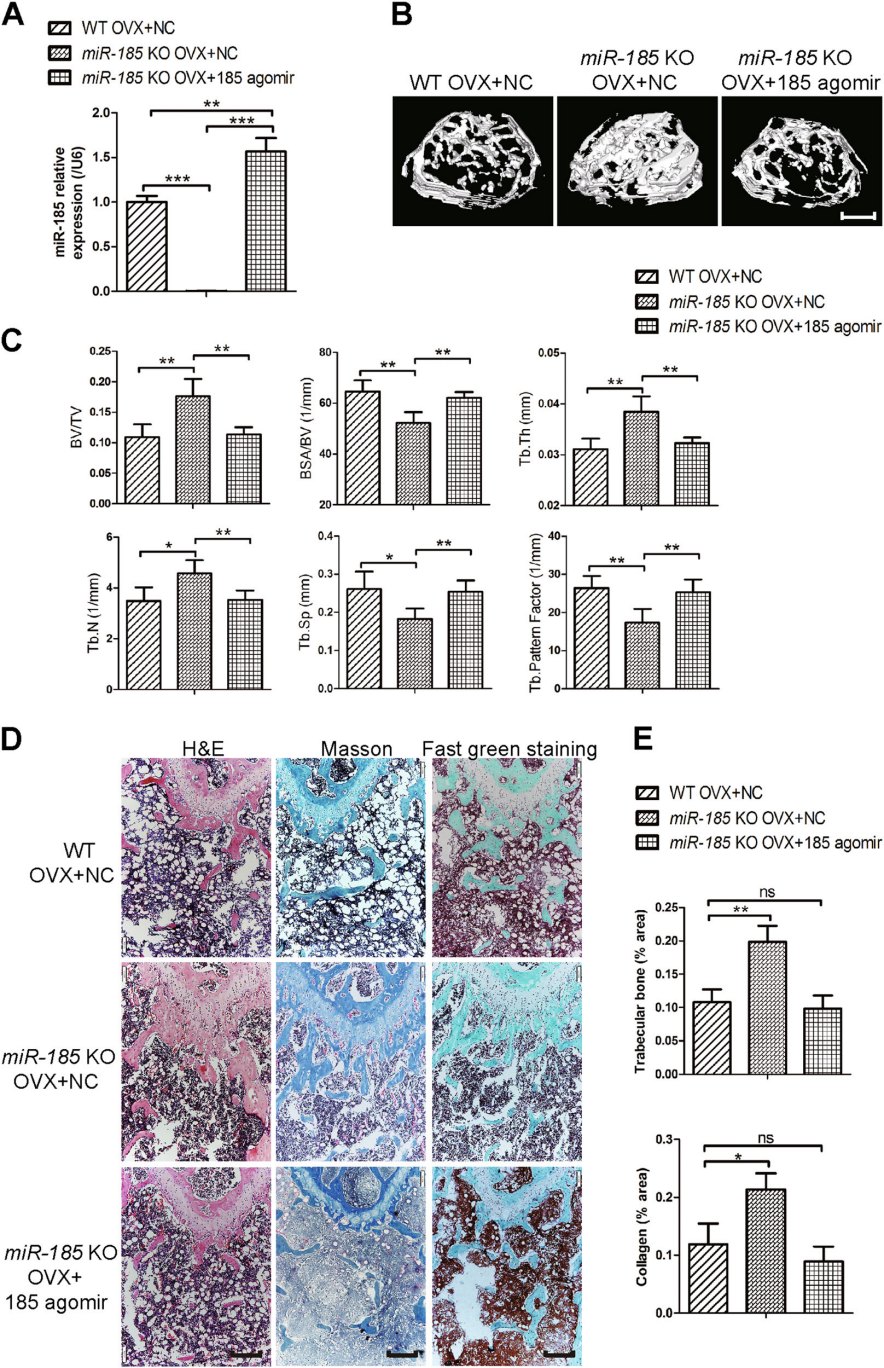
MicroRNA (miR)-185 restoration reverses the bone protection phenotype in osteoporosis of knockout (KO) mice. ovariectomized (OVX)-operated mice were divided into three groups: WT mice with negative control (NC) injection after OVX operation (WT OVX + NC), KO mice with NC injection after OVX operation (*miR-185* KO OVX + NC), and KO mice with miR-185 agomir injection after OVX operation (*miR-185* KO OVX + 185 agomir). Six weeks later, the mice were sacrificed and the bones were harvested. **a** The *miR-185* expression in bones was examined by real-time PCR (*n* = 3). **b** Three-dimensional (3D) reconstructive images of trabecular bones were shown. Scale bar = 400 μm. **c** The microarchitecture of femur epiphysis were analyzed by μCT (*n* = 5). **d** Representative images of hematoxylin and eosin (H&E), Masson’s trichrome staining, and Fast green staining of the femurs in three groups were shown. Scale bar = 200 μm. **e** The trabecular bone area and collagen area were analyzed using the Image J program. Data were shown as mean ± S.D (NS, not significant, *P < 0.05, **P < 0.01, ***P < 0.001)

Because *miR-185* depletion caused partial blockage of bone loss during osteoporosis, we wondered whether restoring it in KO mice could reverse this phenotype in vivo. Mice were injected intravenously with *miR-185* agomir or NC in the first and fourth week after OVX and divided into three groups: OVX WT mice injected with NC, OVX KO mice injected with NC, and OVX KO mice injected with *miR-185* agomir. Bone samples were harvested 6 weeks later. Real-time PCR showed that *miR-185* expression was upregulated in bone samples from agomir-injected KO mice compared to expression levels in WT or KO mice injected with NC (Fig. 5a), which implies successful *miR-185* restoration in agomir-injected KO mice. According to μCT results, Tb. N. and Tb. Th., were significantly lower in KO mice injected with *miR-185* agomir compared to those injected with NC, and there were no obvious differences between *miR-185*-injected KO mice and NC-injected WT mice (Fig. 5b, c).

In addition, the collagen area indicated by Masson’s trichrome staining and the bone area indicated by fast green staining were both reduced after *miR-185* restoration in KO mice (Fig. 5d, e). Taken together, these results further indicate that *miR-185* induces bone loss during osteoporosis.

### *MiR-185* inhibits Bgn expression

**Fig. 6:**
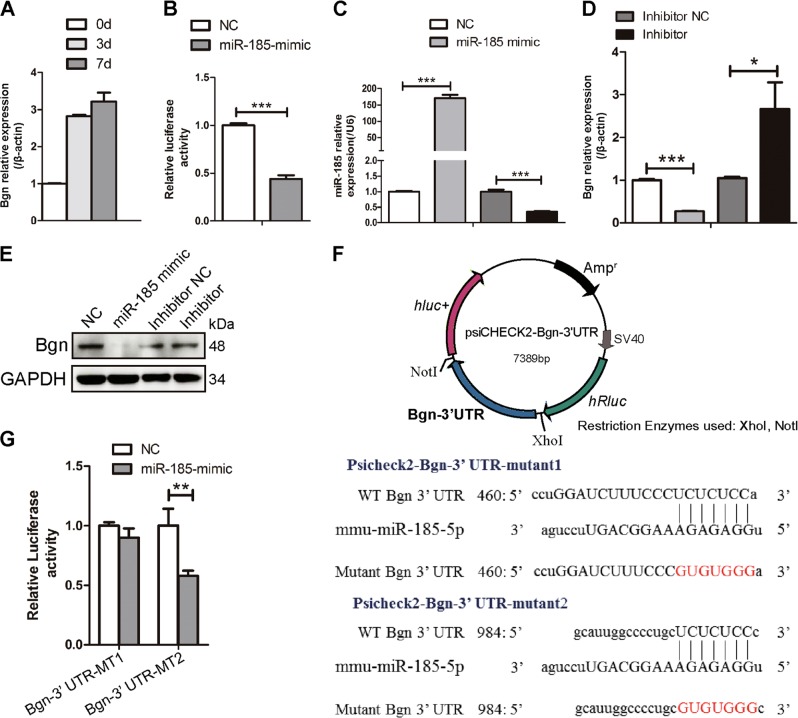
Biglycan (Bgn) is the direct target of microRNA-185 (miR-185). **a** Osteoblasts were cultured with osteoblast induction medium (OIM) for the indicated number of days, and Bgn expression was examined by real-time PCR (*n* = 3). **b** Luciferase activity was detected 48 h after transfection. **c**, **d** MiR-185 mimic and negative control (NC), miR-185 inhibitor and inhibitor NC were transfected into MC3T3-E1 cells, and the messenger RNA (mRNA) expressions of miR-185 and Bgn were examined using real-time PCR (*n* = 3). **e** Forty-eight hours after miRNA transfection, the protein level of Bgn in MC3T3-E1 cells was detected by western blot. **f** Construction of psiCHECK2-Bgn-3′-UTR-WT or mutant plasmids. Schematic of the potential target sites in mouse Bgn 3′-UTR were shown, and two kinds of mutations were generated in the miR-185-5p complementary sites in the reporter vector as indicated. **g** Normalized luciferase activity of the two mutant reporter vectors after miR-185 overexpression. Data were shown as mean ± SD (*P < 0.05, **P < 0.01, ***P < 0.001). UTR, untranslated region, WT wild type.

To elucidate the physiological mechanism underlying *miR-185*-mediated regulation of bone metastasis, we searched for candidate target genes using miRNA target prediction databases and identified *Bgn*, which was first identified in bone matrix and plays a crucial role in osteogenesis^11,12^. Bgn mRNA levels exhibited gradual upregulation during osteoblast differentiation (Fig. 6a). Luciferase reporter assays were carried out using a psiCHECK2-Bgn-3′-UTR construct to investigate whether *miR-185* directly regulates Bgn expression. *MiR-185* mimic or NC was transfected into HEK293T cells with luciferase reporter constructs. The luciferase activity of Bgn 3′-UTR was significantly downregulated by *miR-185* mimic (Fig. 6b). We also conducted experiments in MC3T3-E1 cells to confirm the regulatory role of *miR-185*. Bgn mRNA levels sharply decreased after transfection with *miR-185* mimic (Fig. 6c, d). Meanwhile, the *miR-185* inhibitor significantly increased Bgn mRNA expression compared to the NC (inhibitor NC). Bgn protein levels were also reduced after *miR-185* overexpression (Fig. 6e). These results suggest that *miR-185* inhibits the expression of Bgn during osteoblast differentiation.

The MiRDB database predicted two potential binding sites of *miR-185*-5p in the 3′-UTR of Bgn mRNA. To determine which site is directly recognized by *miR-185*, luciferase reporter plasmids containing mutant recognition sequences were constructed (Fig. 6f). When the first potential binding site was mutated (psiCHECK2-Bgn-3′-UTR-mutant1), *miR-185* failed to downregulate Bgn expression, whereas when the second potential site was mutated (psiCHECK2-Bgn-3′-UTR-mutant2), *miR-185* still lowered the luciferase level (Fig. 6g). Therefore, *miR-185* repressed Bgn gene expression by directly binding to the first seed region in the 3′-UTR of Bgn mRNA.

The Bgn expression was detected in calvarial osteoblasts derived from WT or KO mice. In WT cells, Bgn was slightly expressed at day 0 of osteoblastic induction, and gradually upregulated on days 3 and 7. However, KO cells showed a high expression level of Bgn throughout all time points (Fig. S2A). We also detected the expression of Bgn in MSCs after OIM induction for 4 days, and the result indicated an increased Bgn expression in KO MSCs compared to WT cells (Fig. S2B). In addition, the elevated Bgn expression was also observed in femurs of KO mice compared to WT mice (Fig. S2C).

### *miR-185* depletion enhances BMP/Smad signaling by targeting Bgn

**Fig. 7:**
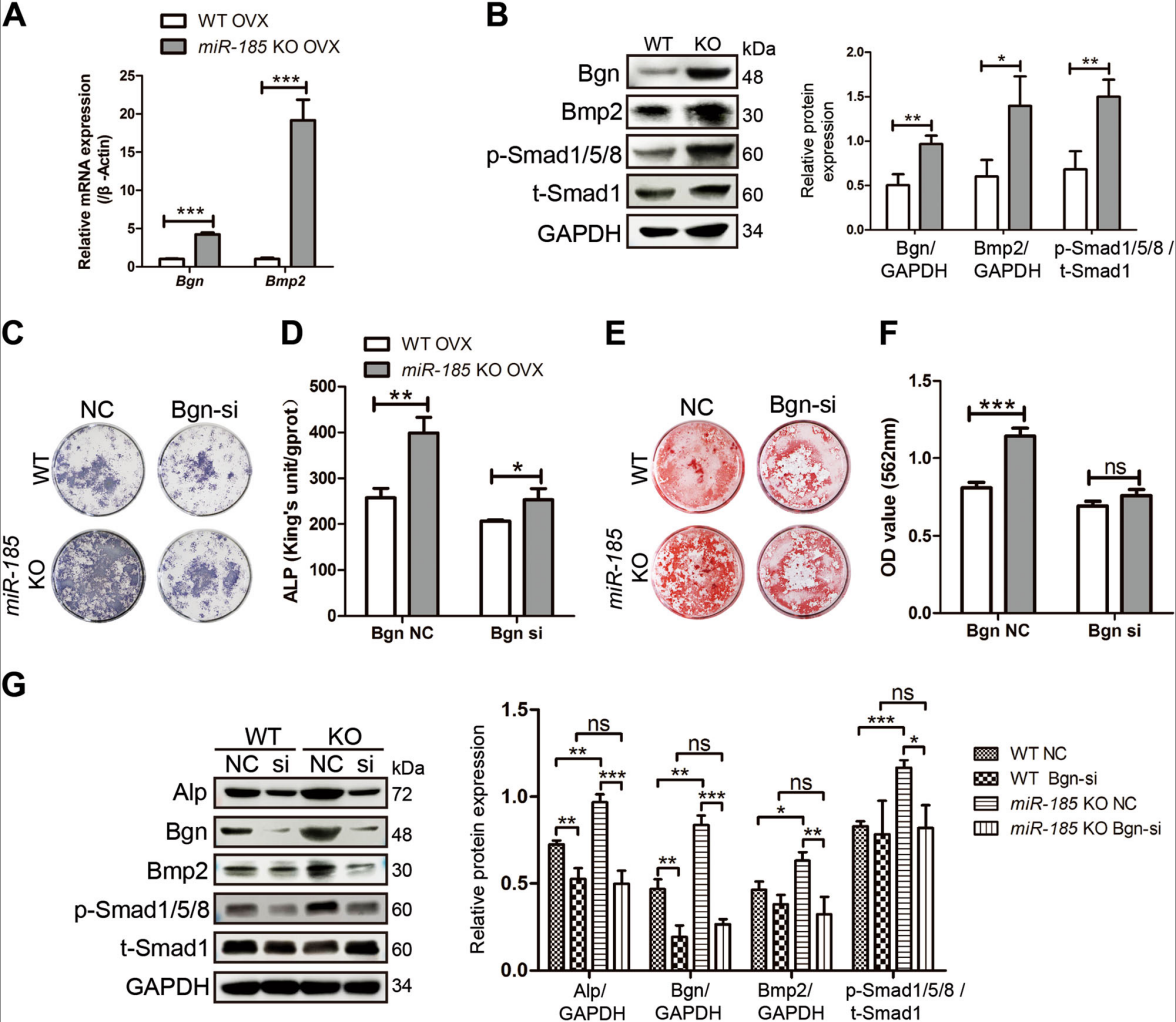
BMP/Smad signaling is regulated by microRNA-185 (miR-185). **a** Six weeks after ovariectomized (OVX) operation, the mice femurs were dissected and total RNA was extracted. The expression level of Biglycan (Bgn) and Bmp2 were determined by real-time PCR (*n* = 3). **b** The expression of Bgn, Bmp2, t-Smad1, p-Smad1/5/8 in wild-type (WT) or knockout (KO) bones after OVX was detected by western blot. **c**, **d** Mesenchymal stem cells (MSCs) were derived from WT or *miR-185* KO mice 6 weeks after OVX operation, and transfected with Bgn small interfering (siRNA) or negative control (NC). Cells were cultured with osteoblast induction medium (OIM) for 7 days and alkaline phosphatase (ALP) staining (**c**) and quantification (**d**) were carried out. **e**, **f** MSCs were derived from OVX mice, and cultured with OIM. Bgn siRNA or NC transfection was carried out at day 0 and day 7. Alizarin Red S staining (**e**) and mineralization quantification (**f**) were performed at day 14. **g** The expressions of Alp, Bgn, Bmp2, t-Smad1, p-Smad1/5/8 in MSCs were detected by western blot after OIM induction for 4 days, and expressed as densitometry normalized to GAPDH. Data were shown as mean ± SD (ns, not significant, *P < 0.05, **P < 0.01, ***P < 0.001)

BMP/Smad signaling plays an important role during bone formation and homeostasis, and studies have shown that the GAG chains of Bgn act as a promoter in Bmp4-induced osteoblast differentiation of murine calvarial cells^12^. In addition, the effector domain of Bgn dramatically increased BMP2 osteogenic function and Smad1/5/8 phosphorylation in C2C12 cells^11^. As indicated by real-time PCR and Western blotting data, the expression levels of Bgn in the bone extracts of KO mice were higher than those in WT mice 6 weeks after OVX (Fig. 7a, b). We assessed whether BMP/Smad signaling was affected in KO mice after Bgn elevation. After OVX, the increased Bmp2 mRNA levels in KO bones compared to WT bones were identified by real-time PCR. Moreover, Western blotting revealed enhanced phosphorylation of Smad1/5/8, which confirmed the activation of BMP/Smad signaling in KO mice in osteoporosis (Fig. 7a, b).

**Fig. 8:**
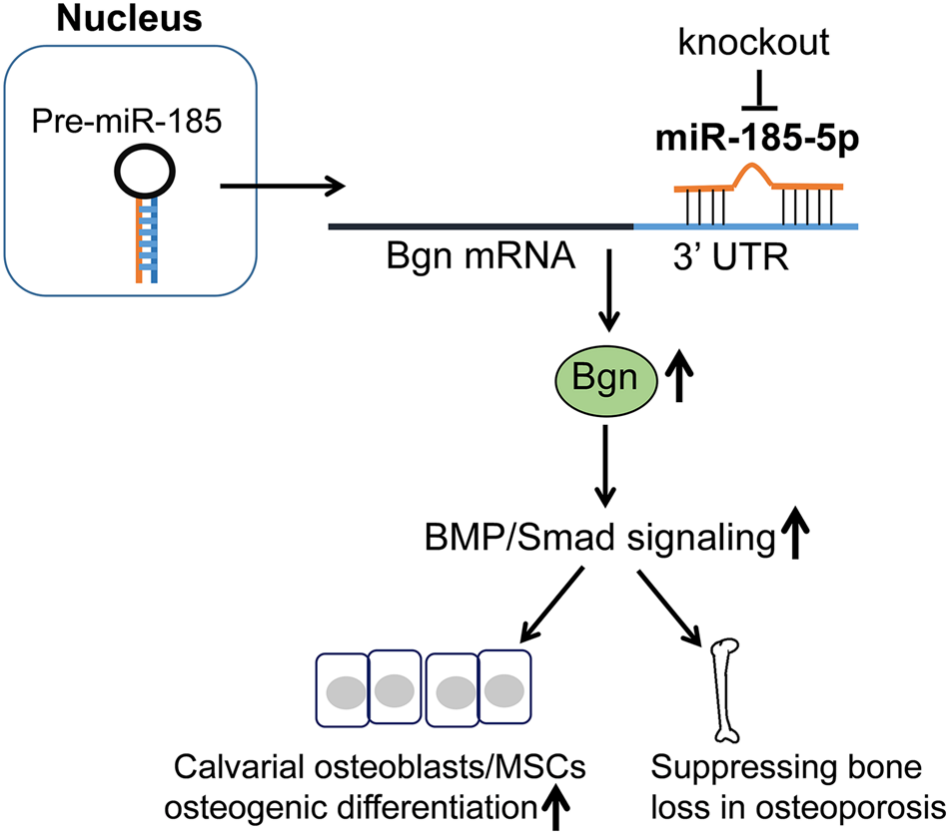
Schematic representation of the miR-185/Bgn/BMP-Smad1/5/8 signaling. In this mechanism, during the osteogenic differentiation, the depletion of *miR-185* upregulated Bgn expression, so as to activate the BMP/Smad1/5/8 signaling. The calvarial osteoblasts and mesenchymal stem cells (MSCs) of knockout (KO) mice exhibited increased osteogenic differentiation ability, and KO mice was protected during osteoporosis. miR-185, microRNA-185; Bgn, biglycan

To further verify the effects of Bgn on the increased BMP/Smad signaling induced by *miR-185* deficiency, MSCs were derived from OVX mice and transfected with Bgn siRNA or NC. Consistent with previous findings, in NC-transfected cells, KO MSCs exhibited increased osteogenic function and mineralization, as indicated by ALP and Alizarin Red S staining and quantification (Fig. 7c–f). Bgn siRNA transfection inhibited osteoblast differentiation in both WT and KO MSCs compared to NC transfection. However, Bgn knockdown partially diminished the pro-osteogenic effects induced by *miR-185* deficiency, as Bgn siRNA transfection reduced the differences between WT and KO cells. These findings were also evidenced by Western blotting data (Fig. 7g). Bgn knockdown blocked the upregulation of ALP, Bmp2, and p-Smad1/5/8 induced by *miR-185* deletion in KO MSCs compared to levels in WT MSCs. Taken together, these data suggest that *miR-185* depletion elevates BMP/Smad signaling, which may be in part through the regulation of Bgn (Fig. 8).

## Discussion

An increasing body of evidence suggests that *miR-185* is a key regulator in bone homeostasis. *MiR-185*-5p was upregulated in Runx2-mutant cells, and inhibited osteogenesis in MC3T3-E1 cells^6^. Downregulation of *miR-185* increased osteoblast viability and decreased cell apoptosis, whereas *miR-185* mimic inhibited cell growth and proliferation^13^. In addition, *miR-185* modulated the expression of tumor growth factor (TGF-β1) after ankle fracture within 2 weeks, indicating a role in fracture recovery in patients^14^.

In this study, we generated *miR-185* deletion mice for the first time, and examined osteogenesis in the KO mice. Primary osteoblasts and MSCs derived from the KO mice exhibited increased differentiation and mineralization compared to cells from their WT littermates. Further, we constructed an OVX mouse model to investigate whether *miR-185* modulates bone homeostasis in vivo. *MiR-185* was upregulated during osteoporosis, and μCT revealed a smaller decrease in bone mass in KO mice than in WT mice. Increased expression of osteoblast markers was also confirmed by real-time PCR. After restoring *miR-185* through agomir injection, the bone-protective phenotype of KO OVX mice was completely reversed. Moreover, we identified Bgn as a direct target of *miR-185*, through which *miR-185* may regulate BMP/Smad signaling during osteoporosis.

The development of osteoporosis is related to an imbalance in the bone microenvironment, which involves the cooperation of many kinds of cells, such as bone marrow stromal cells, osteoblasts, osteoclasts, and adipocytes. The dysregulation of miRNAs in osteoporosis in animals and humans has been demonstrated^15^, and mounting evidence suggests that miRNAs may have therapeutic potential^16,17^. Intravenous injection with chemically synthesized antagomirs of miR-106b-5p, mir-17-5p, miR-451, or miR-103a reversed bone destruction and improved bone strength in mice with OVX-induced osteoporosis^18–20^. In addition, OVX mice injected with chitosan (CH, an effective small nucleotide oligo delivery system) nanoparticles containing miR-182 inhibitors exhibited reduced bone erosion^21^. Because *miR-185* depletion increased BV in OVX mice, we speculate that administration of *miR-185* antagomirs may exert the same pro-osteogenic effects in osteoporosis, and may have therapeutic implications for treating osteolytic diseases.

Bgn is an extracellular matrix proteoglycan in the SLRP family, and is highly expressed in bone and skeletal connective tissues. It has been shown to play a critical role in modulating skeletal development. *Bgn*-depleted mice display decreased growth and reduced bone mass^22^, and *Bgn* mutation has been identified in X-linked spondyloepimetaphyseal dysplasia in humans^23^. Previous studies have shown that Bgn interacts with TGF-β, Bmp2/4, and Wnt signaling and promotes osteogenesis^24–26^.

Bmps have long been identified as important players in bone formation that can induce the differentiation of pluripotent MSCs into osteogenic cells. Bmps bind to receptors and recruit Smad1, Smad5, and Smad8, which then translocate to the nucleus to regulate downstream gene expression^27^. Bmp2 can stimulate osteoblast differentiation and enhance their function. During osteoblast differentiation, Bgn activates Smad1/5/8 signaling, which increases osteoblast differentiation and mineralization in both MC3T3-E1 and C2C12 cells^11,28^. In this study, we demonstrated that *miR-185* regulated Bgn expression, and deletion of *miR-185* elevated Bgn expression during osteoporosis, through which the BMP/Smad signaling pathway was activated, resulting in increased bone mass.

In summary, we demonstrated that *miR-185* can fine-tune osteoblast differentiation and mineralization, and *miR-185* deficiency resulted in reduced bone mass loss during osteoporosis in mice. Our results reveal a previously unrecognized regulatory network mediated through the *miR-185*-Bgn-BMP/Smad axis in osteoblastogenesis, suggesting that *miR-185* inhibition may have therapeutic osteoprotective potential.

## Supplementary information

Figure S1

Figure S2

Figure legend

Supplemental figure legends
