# Supplementary figures and images for: Mmu-miR-185 depletion promotes osteogenic differentiation and suppresses bone loss in osteoporosis through the Bgn-mediated BMP/Smad pathway
Source: Cell Death Dis. 2019 Feb 20;10(3):172. doi: 10.1038/s41419-019-1428-1 (PMC6382812; doi:10.1038/s41419-019-1428-1)

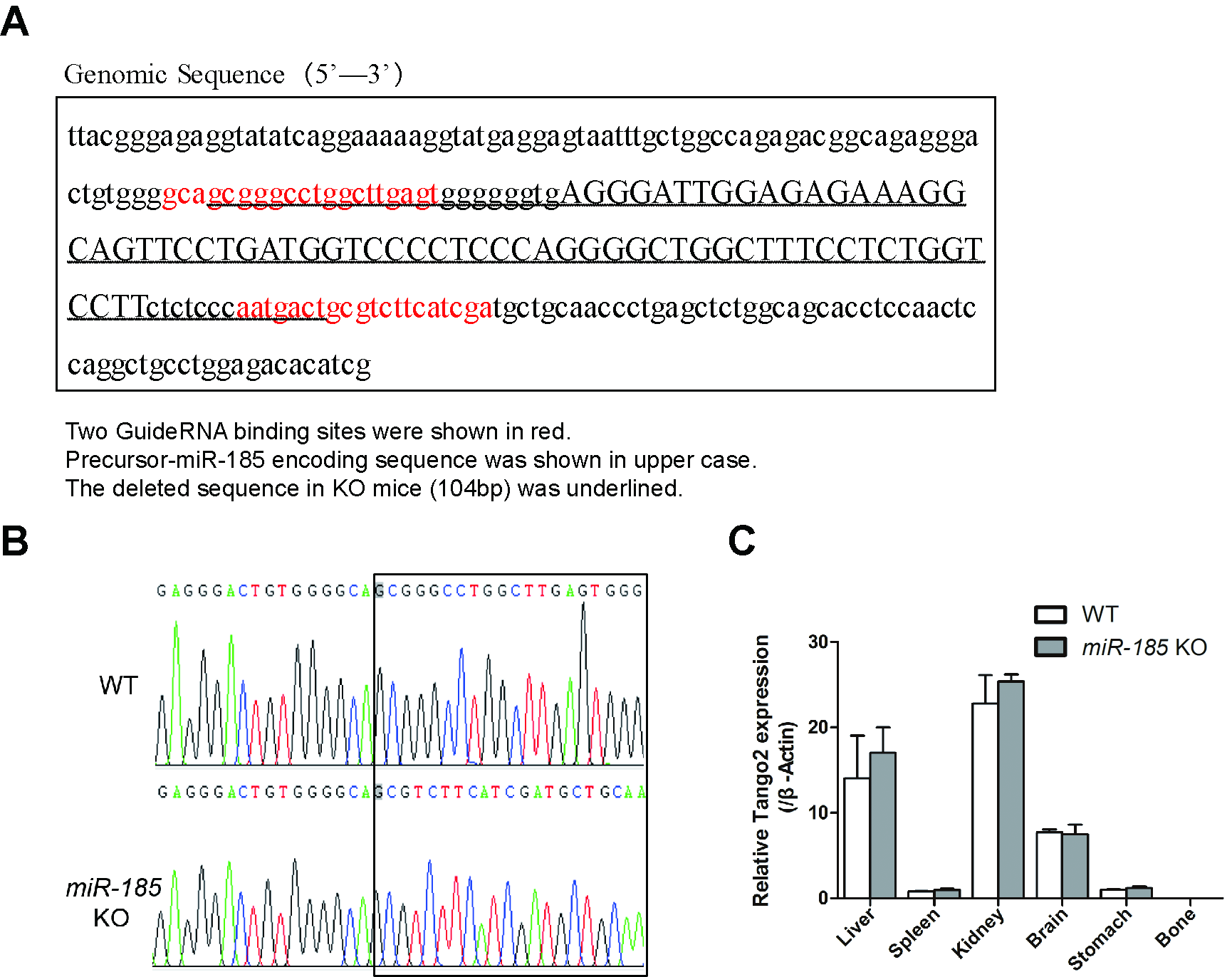

Supplement: Supplementary file 1 — Figure S1 [file 41419_2019_1428_MOESM1_ESM.tif]

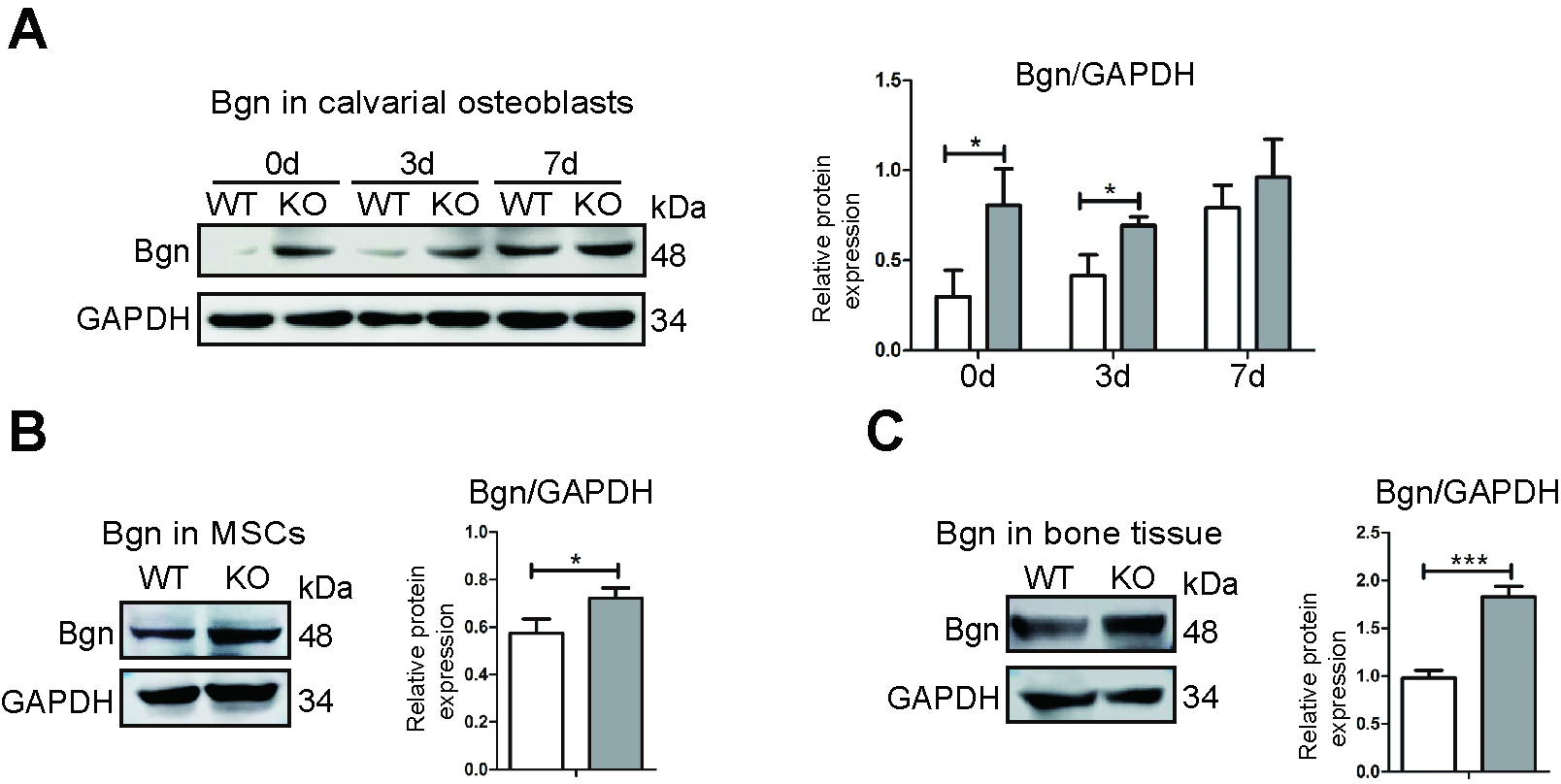

Supplement: Supplementary file 2 — Figure S2 [file 41419_2019_1428_MOESM2_ESM.tif]
