# Supplementary material for: Mmu-miR-185 depletion promotes osteogenic differentiation and suppresses bone loss in osteoporosis through the Bgn-mediated BMP/Smad pathway
Source: Cell Death Dis. 2019 Feb 20;10(3):172. doi: 10.1038/s41419-019-1428-1 (PMC6382812; doi:10.1038/s41419-019-1428-1)
Supplement: Supplementary file 3 — Figure legend [file 41419_2019_1428_MOESM3_ESM.docx]

**Figure S1. Construction of *mmu-miR-185* -/-mice.**

**A.** Genomic sequences of *miR-185* in WT and *mmu-miR-185* KO mice. CRISPR/Cas9 technology caused a 104 bp depletion in *Tango2* intron, which covered the whole coding sequence of *pre-miR-185* (65 bp, letters in uppercase). **B.** Identification of mutations in KO mice (marked by the black frame). **C.** The expression of Tango2 mRNA in different tissues of WT and KO mice (n=3).

**Figure S2. Expression of Bgn *in vivo* and *in vitro*.**

**A.** WT or *miR-185* KO calvarial osteoblasts derived from neonatal mice were cultured with OIM for 0d, 3d, 7d, and western blot analysis were conducted to detect the Bgn expression. **B.** MSCs were derived from the bone marrow of 2-month-old WT/KO male mice, and cultured with OIM for 4 days. Bgn expression was detected. **C.** Bgn expression in femur tissue harvested from 2-month-old WT/KO male mice, and expressed as densitometry normalized to GAPDH. Data were shown as mean±S.D.
